# Supplementary material for: High-throughput Interpretation of Killer-cell Immunoglobulin-like Receptor Short-read Sequencing Data with PING
Source: PLoS Comput Biol. 2021 Aug 2;17(8):e1008904. doi: 10.1371/journal.pcbi.1008904 (PMC8360517; doi:10.1371/journal.pcbi.1008904)
Supplement: S1 Text — (DOC) [file pcbi.1008904.s014.doc]

**S1 text. Genotype determination supporting methods**

Alignment processing workflow

Due to the general difficulty of accurately interpreting multiple-sequence per gene alignments, we built a custom alignment processing pipeline. For exhaustive alignments, reads mapping uniquely to a gene or major allelic group are selected for genotype determination processing. Cross-mapped reads, reads that align to multiple genes or major allelic groups, are sent through a cross-mapped read processing workflow termed readBoost, detailed below. Any cross-mapped read alignments rescued by readBoost processing are added back to the uniquely-mapped read set. After uniquely-mapped reads are selected, the reads are formatted and indexed according to the aligned read formatting procedure, detailed below, before being processed for genotype determination.

For non-exhaustive alignments, all aligned reads are formatted and indexed according to the aligned read formatting procedure before being processed for genotype determination.

Cross-mapped read processing

An issue encountered when processing exhaustive alignments to multiple-sequence per gene references is dealing with reads that map to multiple genes. This issue is especially pronounced for *KIR* short-read sequencing data, where the high sequence similarity between genes can mean that a large proportion of the overall sequencing data can end up cross-mapped. To address this issue, we have developed a cross-mapped read processing method termed readBoost.

If readBoost is enabled, cross-mapped read alignments are processed to rescue reads for use in genotype determination, improving the depth of aligned SNPs. In this process, the alignment score sum for each read-pair is calculated for each mapped reference, subsequently, all reference mappings scoring below the max sum minus a buffer (buffer default is 2) are dropped. All reads that map uniquely to a gene or major allelic group after the poorly scoring reference mappings are dropped are added to the unique alignments to be passed along through the pipeline.

Aligned read formatting

The alignment Concise Idiosyncratic Gapped Alignment Report (CIGAR) (1) string is processed to add any deletion positions marked in the CIGAR string to the read. Additionally, any insertion positions are removed from the read and set aside. Deletions in the reference across the mapped coordinates are added to the reads, and the alignment position is adjusted by the number of preceding deletion positions in the reference to properly scaffold the read in the overall gene alignment. An indexed read vector is generated based on the adjusted alignment position to mark where each nucleotide lies in the overall gene alignment. Finally, previously set aside insertions are added to the corresponding position in the indexed read. If an insertion is followed by subsequent deletion positions, the insertion sequence is written over the deletion characters in the indexed read. Any overhang is saved to the last written position.

The result of this processing is that read alignments to any reference sequence from the same gene are all scaffolded in the same overall gene alignment, enabling processing of these alignments as if a single reference sequence was used.

**References**

1. Li H, Handsaker B, Wysoker A, Fennell T, Ruan J, Homer N, et al. The Sequence Alignment/Map format and SAMtools. Bioinformatics [Internet]. 2009 Aug [cited 2021 Mar 11];25(16):2078–9. Available from: /pmc/articles/PMC2723002/
