# Supplementary material for: High-throughput Interpretation of Killer-cell Immunoglobulin-like Receptor Short-read Sequencing Data with PING
Source: PLoS Comput Biol. 2021 Aug 2;17(8):e1008904. doi: 10.1371/journal.pcbi.1008904 (PMC8360517; doi:10.1371/journal.pcbi.1008904)
Supplement: Attachment — Submitted filename: PING_Reviewer_response.docx [file pcbi.1008904.s015.docx]

We wish to thank the reviewers for taking the time to review this manuscript and for their thoughtful and constructive critiques. We have responded to their specific points (grey) below (black). All modifications have been tracked in the text of the manuscript.

Reviewer #1: Authors describe a new version of the PING tool for genotyping of killer cell immunoglobulin-like receptor (KIR) genes that are critically important in immunogenomics studies and applications. Authors benchmark PING on many reference datasets and demonstrate its accuracy. While I am positive that this manuscript will present an interest for readers of PLOS Computational Biology, I highly recommend addressing the following comments regarding the description of the pipeline and benchmarking against the existing tools prior to publication:

1. I find the placement of the “K-mer similarity analysis” section confusing. Seems like the purpose of this section is to introduce the definition of shared k-mers that is used for demonstration of high repetitiveness of KIR genes, and the k-mer analysis itself is not a part of the PING tool. If so, I recommend combining this section with the section in Results as the definition of shared k-mers is pretty straightforward and probably shouldn’t take the whole section of the Methods.

**We agree with the reviewer that the manuscript will be improved by this reorganization. In the revised manuscript, Methods and Results have been amended in accordance with this recommendation.**

2. It is difficult to create the correspondence between pipeline blocks in Figure 1 and sections of the Methods. Could you please add names of blocks from Figure 1 to the titles of sections to make understanding the pipeline easier?

**We have revised the Method section titles in accordance with this suggestion.**

3. Sections describing preprocessing of the KIR gene database and the pipeline are currently intermixed - e.g., the “Copy number determination workflow” section corresponding to the pipeline is located between sections describing manipulations with the database. Since it complicates following the Methods, I recommend making a separate section “Preprocessing the database” and make sections “Imputation of uncharacterized regions and extension of untranslated regions to generate comprehensive alignment reference sequence” and “Designing a minimized reference allele set” its subsections.

**We thank the reviewer for this suggestion. We have revised Methods to include these improvements.**

4. Line 223: initial genotyping and final genotyping are not introduced before and their purposes are unclear. Are they explained later in lines 274-289? In any case, add a short description of these steps before using them.

**We apologize for this oversight and thank the reviewer for this suggestion. We have added text to describe these steps in the revised manuscript.**

5. The overall description of the PING pipeline largely overlaps with the description provided in Norman et al., AJHG, 2016. Please provide a more specific description of the differences from the previous work in addition to one provided in lines 88-98.

**In order to provide the reader with a complete description of the pipeline, we describe it fully, including elements previously described in Norman et al. However,** **this work expands and improves on the pipeline described in Norman et al. in many important ways: the copy number determination workflow was adjusted from a single-sequence per gene alignment to a comprehensive multiple-sequence per gene alignment; virtual probes used for gene content determination were refined and expanded; the genotype determination workflow was adjusted from static single-gene filtration alignments to dynamic holistic alignments, which incorporate the so-established gene content and preliminary genotype determinations; a custom alignment processing workflow was developed to handle multiple-sequence per gene alignments; and finally, a *KIR* sequence imputation workflow was developed to enable alignment to any described *KIR* allele sequence. In the revised manuscript, we briefly outline these details in order to better orient the reader to make clear that the pipeline is substantially different from that described in Norman et al.**

6. Could you please comment on using long read technologies for genotyping and haplotyping KIR loci? Considering their relatively small sizes, I can imagine that a 70 kbp locus can be completely spanned by a single Pacbio / Nanopore read. Have you tried to use long read data in addition to short reads?

**Long read data would be beneficial for phasing, potentially improving genotype ambiguity and haplotype identification.** **While long read data from a platform with cost-effective methods like nanopore might be difficult to interpret due to the high error rates, the combination of long reads and short reads would cover the weaknesses of the respective technologies and should provide a highly accurate KIR interrogation method; indeed, long read methods have provided valuable insights into KIR haplotypes (eg Roe et al.). We have not had the opportunity to test long read technologies, but we anticipate a need for careful consideration of potential read misalignments when aligning the short and longs reads together. Higher fidelity methods, such as PacBio, are currently under development, but currently the cost is prohibitive for the kind of high-throughput studies that PING was designed to address.**

7. Have you benchmarked the new version of PING against recently released tools by Jieming Chen et al., Briefings in Bioinformatics, 2020 (doi.org/10.1093/bib/bbaa223) and Roe and Kuang, Front Immunol, 2020 (doi.org/10.3389/fimmu.2020.583013)? Please also add citations of these papers to the Introduction.

**We appreciate the suggestion to consider other tools intended to support *KIR* genotyping. We have added text to the Introduction to comment on the existing *KIR* typing methods KPI and KIR*IMP. We note these tools are designed to interrogate *KIR* for gene content only, not the high-resolution allelic genotyping (in addition to gene content and copy number) that PING has been designed to support.**

**Per the referee’s suggestion we have now benchmarked PING against KPI (Roe and Kuang) for determining KIR gene content, achieving 100% concordance for *KIR3DP1*, *KIR2DL3*, *KIR2DL4*, *KIR3DL3* and *KIR3DL2*, over 97% concordance for *KIR2DS5*, *KIR2DP1*, *KIR2DS3*, *KIR2DS2*, *KIR3DL1*, *KIR2DL2*, *KIR2DS4*, *KIR2DL1*, and *KIR2DL5*, and over 95% concordance for *KIR3DS1*, and *KIR2DS1*. We describe these results in the revised manuscript.**

Reviewer #2: The manuscript describes a new tool for determination genotype and copy number for KIR genes. The tool is of huge interest and beautifully tackled the reference alignment part. It is also impressive to see that the tool is further applied to real-life scenario to see KIR genes copy number in population cohorts.

Strengths:
The manuscript stick to the theme in defining the problem and presenting a viable solution to it.
The underlying codes and reference sequences are made available through github repo.

Comments:
1. The workflow is interesting, however there seems to be no source of ground truth. Since, the authors have validated it in a simulated data, how we can be sure about the accuracy values?

**This is a significant issue for *KIR* methods development as the extensive sequence variation and overall homology of the region have been a barrier to widespread adoption of high-resolution sequencing methods. Bioinformatic sequence analysis tools are limited to the direct analysis of gene content with copy number determination possible through imputation, and biochemical methods are either limited to the detection of particular alleles or lack the necessary resolution, presenting substantial roadblocks to producing a robust ground truth dataset.**

**To provide confidence in the efficacy of the workflow we have provided three distinct validation datasets. Our European dataset was both manually curated and independently sequenced and genotyped by our collaborator, DKMS, providing additional confidence in the ground truth genotypes. The synthetic dataset was constructed to challenge the workflow with a diverse allele reservoir and extensive copy number variation based on randomly combining observed *KIR* haplotypes and provides an absolute source of ground truth. Finally, we have manually curated a Khoesan dataset to provide a highly divergent and complex test population. Together, the performance of these datasets show that our workflow accurately and robustly interprets *KIR* sequencing data.**

2. While determining the genotypes, the authors are detecting the individual alleles and there are a few tools available for determine alleles in KIR data, the authors have not presented any comparison or references to those tools.

**To the best of our knowledge there are two existing publicly available tools for interrogating *KIR* genomic data, KIR*IMP and KPI. KIR*IMP imputes *KIR* copy number from carefully selected SNPs while KPI interprets *KIR* gene content and predicts haplotype-pairs using *in silico* probes. Neither of these methods support allele level genotyping or direct copy number assessment, which are the main advancements enabled by PING. We benchmarked PING against KPI (Roe and Kuang) for determining *KIR* gene content, achieving 100% concordance for *KIR3DP1*, *KIR2DL3*, *KIR2DL4*, *KIR3DL3* and *KIR3DL2*, over 97% concordance for *KIR2DS5*, *KIR2DP1*, *KIR2DS3*, *KIR2DS2*, *KIR3DL1*, *KIR2DL2*, *KIR2DS4*, *KIR2DL1*, and *KIR2DL5*, and over 95% concordance for *KIR3DS1*, and *KIR2DS1*. We describe these results in the revised manuscript.**

3. It is shown in the Figure 4 that even with 250-mer, few of the genes shared a high percentage with related genes. In such a case where the short read size is around 250 bases, how come any reliable selection of gene could be made?

**This is the rationale behind making an initial gene content determination, which can still be reliable even for genes with high degrees of shared sequence, then, using the determined gene content to inform subsequent alignments so absent genes are not represented in the alignment reference, limiting the search space of potential shared sequence. This strategy extends to the genotype aware alignments, where an initial genotype determination informs subsequent alignments to further restrict the space of potential shared sequence.**

4. One of the claim in the manuscript is that the reference alignment is improved in the workflow because of using an allele diversity, instead of single gene reference. However, no data is shown for improvement, and whether it is significant.

**We apologize for this oversight and thank the reviewer for this suggestion. To resolve this issue,** **we have performed a comparison of single-sequence per gene vs. multiple-sequence per gene alignments using the synthetic dataset. The reads from this dataset are labeled according to their source gene, providing a straightforward approach to quantify alignment improvement. This comparison showed substantial reductions in the frequency of read misalignments across *KIR2DL1*, *KIR2DL23*, *KIR2DL5A*/B, *KIR2DS1*, *KIR2DS35*, and *KIR3DL1S1*, and small reductions for *KIR3DL2*, *KIR3DL3*, and *KIR3DP1* for the multiple-sequence per gene reference. Overall, these results corroborate our empirical observations that including diverse allele sequences in the reference improves alignment fidelity. We describe these results in the revised manuscript.**

5. Genotype aware alignment might be carrying forward some biases and there would be issues in working with novel genotypes. Authors have not commented anything on this point.

**This is an excellent point; alignment biases and novel genotype identification has been an ongoing struggle in developing *KIR* sequence interrogation methods. The *KIR* system in particular is sensitive to reference sequence bias because the** **combination of highly polymorphic genes and high sequence similarity between genes means that small changes in the reference sequence can have large impacts on read alignments.** **This is a problem even for non-genotype aware alignments because all alignments are inherently biased by the reference sequence. We have implemented several methods to counteract potential alignment biases that could be carried forward by the genotype aware alignments. The first is the use of virtual probes to identify alleles and structural variants prone to misidentification. The second is the addition of a curated sequence set to the alignment reference for any gene with a determined genotype that does not perfectly match the aligned SNPs, these sequences were selected to cover a large amount of the allelic diversity of the corresponding gene. The rationale behind this method is that mismatched allele typings are more likely to be caused by misaligned reads than true novel genotypes, and the use of the mismatched allele sequence as a reference will cause the read misalignments to be repeated. Even with these countermeasures we still encounter some improper novel genotype determinations. Since no novel genotypes were simulated for the synthetic data the Synthetic data in the Unresolved column of Table 2 represents improper novel genotype determinations. In the revised manuscript, we note this limitation and potential for bias in Discussion.**
